# Gastrointestinal Mechanisms Underlying the Cardiovascular Effect of Metformin

**DOI:** 10.3390/ph13110410

**Published:** 2020-11-22

**Authors:** Malcolm J. Borg, Christopher K. Rayner, Karen L. Jones, Michael Horowitz, Cong Xie, Tongzhi Wu

**Affiliations:** 1 Adelaide Medical School and Centre of Research Excellence in Translating Nutritional Science to Good Health, The University of Adelaide, Adelaide 5000, Australia; malcolm.borg@sa.gov.au (M.J.B.); chris.rayner@adelaide.edu.au (C.K.R.); karen.jones@adelaide.edu.au (K.L.J.); michael.horowitz@adelaide.edu.au (M.H.); c.xie@adelaide.edu.au (C.X.); 2 Endocrine and Metabolic Unit, Royal Adelaide Hospital, Adelaide 5000, Australia; 3 Institute of Diabetes, School of Medicine, Southeast University, Nanjing 210096, China

**Keywords:** metformin, cardiovascular, gastrointestinal, mechanisms, bile acids, gut microbiota, glucagon-like-peptide-1, gastric emptying, postprandial hypotension, type 2 diabetes

## Abstract

Metformin, the most widely prescribed drug therapy for type 2 diabetes, has pleiotropic benefits, in addition to its capacity to lower elevated blood glucose levels, including mitigation of cardiovascular risk. The mechanisms underlying the latter remain unclear. Mechanistic studies have, hitherto, focused on the direct effects of metformin on the heart and vasculature. It is now appreciated that effects in the gastrointestinal tract are important to glucose-lowering by metformin. Gastrointestinal actions of metformin also have major implications for cardiovascular function. This review summarizes the gastrointestinal mechanisms underlying the action of metformin and their potential relevance to cardiovascular benefits.

## 1. Introduction

Type 2 diabetes (T2D) is a key global health issue with rising prevalence [1]. T2D is associated with a two-to-three-fold risk of cardiovascular disease, which represents the most common cause of death. Anti-diabetic treatment focuses on glucose-lowering which, when effective, markedly diminishes the risk of both development and progression of microvascular complications (i.e., neuropathy, retinopathy, nephropathy). However, improvements in glycemic control only have modest effects on macrovascular outcomes, with only a subset of anti-hyperglycemic agents being associated with a beneficial effect on cardiovascular outcomes in T2D, including sodium glucose linked transporter-2 (SGLT-2) inhibitors and glucagon-like peptide-1 (GLP-1) receptor agonists, along with metformin [2].

Metformin is one of the oldest anti-diabetic drugs and remains the first-line pharmacological therapy for T2D in most international guidelines [3]. Its pleiotropic effects, in addition to glucose-lowering, include anti-obesity, anti-cancer, anti-ageing and cardiovascular benefits. The latter, demonstrated initially in the UKPDS trial, is of increasing interest. In the UKPDS trial, metformin use was associated with a moderate reduction in the incidence of various cardiovascular end-points and all-cause mortality, relative to both diet-only therapy and intensive glucose-lowering with alternate treatments, in overweight T2D patients [4,5]. These cardiovascular benefits were confirmed in a number of subsequent studies [6,7,8,9], although, in a minority of studies, neutral or deleterious cardiovascular outcomes were reported with metformin use in T2D [10,11].

Metformin is an effective glucose-lowering agent in both the fasting and postprandial states. The latter is notable given that postprandial hyperglycemia represents an independent cardiovascular risk factor [12]. Fluctuations in postprandial blood glucose, in particular, are strongly associated with the macrovascular complications of T2D [13], while a rapid rise in postprandial blood glucose is associated with endothelial injury and initiation of a pro-atherogenic cascade [14,15]. It is generally considered, however, that the cardiovascular benefits of metformin are primarily related to extra-glycemic mechanisms. Mechanistic studies have hitherto focused on the direct effects of metformin on the heart and vasculature. A heterogenous array of potential mechanisms have been identified, including changes in left ventricular function, attenuation of ischemic-reperfusion injury, reduction in sympathetic activity and adrenergic receptor down-regulation, improved endothelial function, changes in the renin-angiotensin-aldosterone system, blood pressure lowering and anti-inflammatory effects. Not surprisingly, the mechanisms accounting for cardiovascular benefit with metformin therapy remain contentious [16].

The anti-hyperglycemic mechanisms of metformin have been historically ascribed to the effects mediated following drug absorption, particularly hepatic glucose metabolism [17]. More recently, there has been increasing recognition that the gastrointestinal tract is, at least, of similar importance, to the liver for glucose-lowering by metformin [18,19,20,21]. Notably, a novel delayed-release formulation of metformin, which releases its active component in the lower jejunum and ileum, induces comparable glucose-lowering compared with standard formulations of metformin, despite minimal bioavailability [19,22].

The potential implications of the gastrointestinal actions of metformin for the cardiovascular system have been largely ignored [23]. This review summarizes the gastrointestinal actions of metformin and their potential relevance to its cardiovascular effects in T2D.

## 2. Gastrointestinal Actions of Metformin and Cardiovascular Function

**Figure 1:**
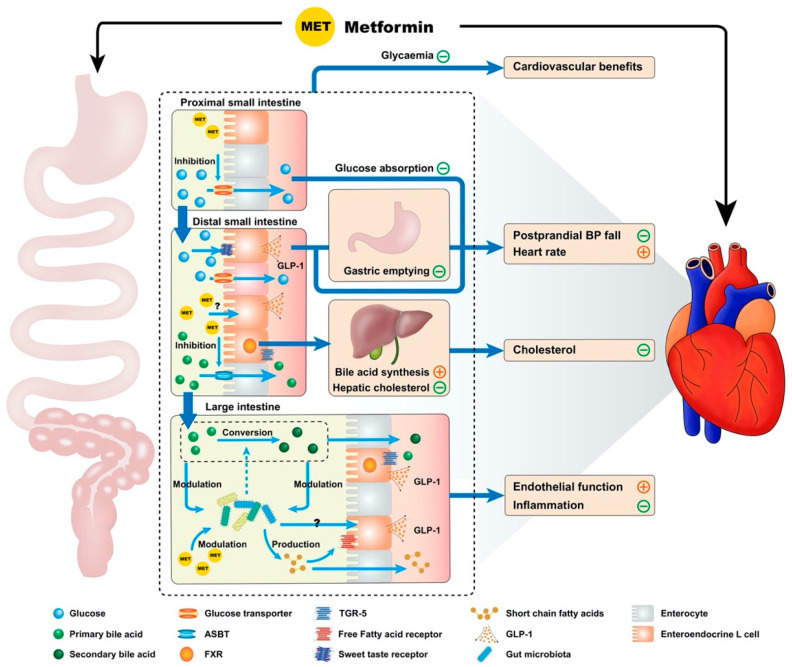
Proposed model by which metformin has cardiovascular actions via gastrointestinal mechanisms. Metformin potentially influences the cardiovascular system through multiple interconnected mechanisms derived from the drug’s interactions with the gastrointestinal tract. These include effects on intraintestinal bile acids (inhibition of resorption, reduction in hepatic low density lipoprotein cholesterol production secondary to reduced enterohepatic bile acid circulation and altered bile acid composition), modulation of the gut microbiota, reduction in the rate of small intestinal glucose absorption, enhanced glucagon-like peptide-1 (GLP-1) secretion and action and delayed gastric emptying. Many of these mechanisms would be expected to modulate the postprandial blood pressure (BP) response, providing a potential therapeutic option for the management of postprandial hypotension. Notable gastrointestinal receptors are referenced above, such as the apical sodium-dependent bile acid transporter (ASBT), farnesoid X receptor (FXR) and Takeda G-coupled receptor 5 (TGR-5).

Metformin exhibits multiple interconnected gastrointestinal effects, which have a potential significance for cardiovascular function, including inhibition of bile acid resorption, modulation of the gut microbiota, reducing the rate glucose absorption, enhanced GLP-1 secretion and action, slowing of gastric emptying and attenuation of the fall in blood pressure after a meal (Figure 1) [23,24].

### 2.1. Inhibition of Bile Acid Resorption

Metformin reduces bile acid resorption in the ileum and, hence, increases fecal bile acid excretion [25,26]. Although bile acids are primarily known for their role in fat digestion, they interact with multiple receptors within, and external to, the gastrointestinal tract [27]. Notably, bile acids activate the membrane Takeda G-coupled receptor 5 (TGR-5) and the perinuclear receptor farnesoid X receptor (FXR). Increased activation of TGR5 and reduced activation of FXR have been associated with inhibition of bile acid resorption [28]. Mechanistically, this may be accounted for by reduced uptake of bile acids by enterocytes, leading to an increase in the intestinal stimulation of membrane-bound receptors and a reduction in perinuclear receptor stimulation. Moreover, altered bile acid composition—secondary to metformin-induced changes in intestinal microbiota—favors production of glycoursodeoxycholic acid (GUDCA), a secondary bile acid which acts as an FXR antagonist [29]. TGR5 receptor activation induces the release of GLP-1 from the enteroendocrine L-cells. That metformin enhances bile acid-mediated GLP-1 secretion is supported by potentiation of cholecystokinin-induced GLP-1 secretion in healthy males receiving metformin therapy sub-acutely [30]. Bile acids may also contribute to metformin-enhanced GLP-1 secretion via their “bitterness”, since enteroendocrine L-cells express a variety of bitter taste receptors [31]. The potential cardiovascular implications of enhanced GLP-1 secretion are discussed later in this review. The FXR receptor is present primarily in organs exposed to high bile acid concentrations, i.e., liver, kidney and gastrointestinal tract, but is also expressed in the arterial vasculature and the heart [32]. Suppression of FXR-signaling may augment GLP-1 secretion [33] and has been reported to promote beneficial cardiac remodeling following myocardial infarction in rodents [34].

Bile acids exert numerous direct effects on the systemic circulation, including negative chronotropy and inotropy, as well as anti-apoptotic effects on cardiac myocytes, increased nitric oxide production, variable effects on angiogenesis and reduced inflammation [35]. While the impact of these effects on cardiovascular function remain uncertain, studies of liver cirrhosis and bile acid sequestrants, a class of anti-diabetic agents that bind to intestinal bile acids to prevent their reabsorption, are indicative of the benefits of a reduction in the systemic accumulation of bile acids. Systemic accumulation of bile acids in patients with cirrhosis is linked with a constellation of negative cardiac effects, which constitute “cirrhotic cardiomyopathy.” In pre-clinical models, these changes have been shown to be reversible with the use of intestinal bile acid sequestrants [36,37]. Furthermore, the lowering of plasma low-density lipoprotein cholesterol (LDL-C) by bile acid sequestrants, such as cholestyramine, reflects depletion of the hepatic bile acid supply, thereby stimulating hepatic metabolism of cholesterol for bile acid synthesis [38]. Given the association of hypercholesterolemia with atherogenesis, it has been assumed that bile acid sequestration would convey significant cardiovascular benefits, but adequately powered studies have shown, at most, a trend towards cardiovascular event reduction, with these agents [38]. Metformin lowers LDL-C levels, which should intuitively reduce cardiovascular risk; however, the effect of metformin to reduce LDL-C is less than bile acid sequestrants (~6.5% with metformin vs 12% with cholestyramine use). Moreover, cholesterol-lowering by metformin may occur via alternate pathways, such as hepatic AMPK activation, in addition to the reduction in bile acid absorption [39,40,41,42].

Interestingly, the elevation in serum bile acid levels, which occurs following all forms of bariatric surgery, with the exception of gastric banding, has been associated with the metabolic benefits of these procedures. It has been suggested that bile acids contribute to the metabolic benefits of bariatric surgery via systemic FXR activation [43]. However, relative to cirrhosis, the increase in serum bile acid concentrations reported following bariatric surgery is modest, e.g., mean fasting serum bile acid concentrations of 8.90 µmol/L, when compared to 110 µmol/L in patients with cryptogenic or alcoholic cirrhosis [44,45,46]. This may indicate that the concentration of systemic bile acids is critical, being cardiotoxic at pathologically high concentrations while conveying cardiovascular benefits at physiologically elevated concentrations.

### 2.2. Modulation of the Gut Microbiota

An ever-increasing body of literature supports the importance of the gut microbiota in health and disease. Shifts in the abundance of numerous micro-organisms have been linked to the pathogenesis of T2D, obesity and cardiovascular disease [47]. Metformin clearly modulates gut microbiota. This effect, which is of uncertain etiology, has been demonstrated in animal models of T2D in which broad-spectrum antibiotics were shown to abrogate the anti-diabetic effect of metformin in high-fat-fed rodents [48]. In humans, use of metformin has been associated with proliferation of numerous short-chain fatty acid-producing bacteria (e.g., *Prevotella*), *Akkermansia muciniphilia* and *Clostridium cocleatum* and decreased the abundance of *Clostridaceae 02d06* and *Bacteroides fragilis* [49,50]. Intestinal short-chain fatty acids, per se, have the capacity to modulate blood glucose and cardiovascular function through inducing weight loss, stimulation of GLP-1 secretion and anti-inflammatory effects [28]. In addition, intestinal *Akkermansia* species may, by degrading mucin, increase the capacity of the intestinal mucosal barrier to reduce translocation of proinflammatory lipopolysaccharides [48]. *Bacteroides fragilis* converts primary bile acids to secondary bile acids and, when reduced, is associated with higher concentrations of the bile acid GUDCA. As discussed, GUDCA is a potent FXR antagonist, through which gut microbiota may contribute to metformin-enhanced GLP-1 secretion. However, the effects of the microbial changes described remain to be demonstrated in long-term clinical studies. The relationships between certain microbiota, metformin use, and disease is complex. For example, both metformin and T2D are associated with increased *Prevotella*, albeit through different operational taxonomic units, i.e., bacteria with contrasting DNA, but below the threshold of genetic variability is to be classed as a separate species [51]. It remains uncertain whether there are variable effects on body metabolism by these taxonomic units. Furthermore, the majority of bacteria implicated in cardiovascular disease are not known to be affected by metformin [49,52]. The efficacy of microbiota-based therapy (e.g., probiotics, prebiotics and fecal microbiota transplantation) for mitigating cardiovascular risk in humans is also uncertain [53]. Further studies are required to consolidate the links between the gut microbiota profile and health outcomes, and to evaluate the efficacy of microbiota-based therapies.

### 2.3. Reducing the Rate of Glucose Absorption

Investigation into the impact of biguanides on intestinal glucose absorption dates back several decades. Caspary and Creutzfeldt, in 1971, identified an inhibitory effect on active intestinal glucose transport by several biguanides in vitro when investigating the potential alternate glucose-lowering mechanisms of metformin [54]. Subsequently, inhibition of intestinal glucose absorption by metformin was demonstrated, albeit inconsistently, in animal studies [55,56,57,58]. Recently, a modest reduction in the rate of glucose absorption was demonstrated in patients with T2D receiving metformin sub-acutely [59]. Glucose transporters at the brush border and serosal surface of enterocytes have been implicated in this effect, including sodium glucose-linked transporter-1, glucose transporter-2 and insulin-sensitive channels [24,55]. Interestingly, when glucose transport across the enterocyte was investigated in vitro, the concentration of metformin within the arterial blood supply appeared to be more relevant than that within the enterocyte, suggesting that systemic absorption of metformin is necessary for inhibition of intestinal glucose absorption [56].

While the magnitude of the effect of metformin on glucose absorption may be modest, the effect may be sufficient to influence the hormonal and hemodynamic responses to carbohydrate. Inhibition of carbohydrate absorption in the proximal small intestine would increase glucose exposure to the GLP-1-secreting enteroendocrine L-cells, located primarily in the ileum and large intestine, thereby potentially enhancing GLP-1 secretion [60]. Moreover, diverting nutrient absorption from the proximal to the distal gut may attenuate the substantial fall in postprandial blood pressure that is often observed in patients with T2D [61]. The implications of enhanced GLP-1 secretion and postprandial hemodynamic changes are discussed in this review subsequently.

The aforementioned hormonal and hemodynamic responses to impaired glucose absorption are expected to contribute to the cardiovascular benefits of metformin given that they have been replicated with the use of acarbose in T2D [62,63,64,65,66]. Acarbose, an alpha-glucosidase inhibitor, acts to delay the breakdown of complex carbohydrates and thus reduces the rate of glucose absorption. In the STOP-NIDDM trial, potential cardiovascular benefits with acarbose therapy was evident—over ~3.3 years there were reductions in the development of hypertension, progression of arterial intima-media thickening (a surrogate marker for coronary atherosclerosis) and cardiovascular events in patients with impaired glucose tolerance [67,68]. In a meta-analysis, acarbose was also shown to delay the occurrence of myocardial infarction and other cardiovascular events in T2D [69]. It has been suggested that the STOP-NIDDM study lacked sufficient power to demonstrate cardiovascular benefits with acarbose due to the low number of cardiovascular events during the study [70,71]. In a recent large randomized, placebo-controlled trial of acarbose, the so-called ACE study, in Chinese patients with coronary heart disease and impaired glucose tolerance, as well as a recent meta-analysis including patients with impaired glucose tolerance or T2D, acarbose was reported to exhibit a neutral impact on cardiovascular events [15,72]. It is, accordingly, likely that inhibition of glucose absorption contributes, but is not fundamental, to the cardiovascular benefits of metformin.

### 2.4. Enhanced GLP-1 Secretion and Action

It is now widely appreciated that metformin has the capacity to increase plasma GLP-1 levels, an effect potentially related to stimulation of endogenous GLP-1 secretion and/or inhibition of dipeptidyl peptidase-4 (DPP-4), the ubiquitous enzyme which breaks down intact GLP-1 [73]. The mechanisms by which metformin stimulates GLP-1 remain incompletely understood and controversial. While in vitro studies using isolated human intestinal biopsies have suggested that metformin may have a direct effect to induce GLP-1 secretion from enteroendocrine L-cells [28], targeted perfusion of metformin alone to the ileum (where L-cells are densely distributed) failed to induce a meaningful change in plasma GLP-1 levels in patients with T2D [74]. In the latter study, acute administration of metformin into either the proximal or distal small intestine one hour prior to an oral glucose load augmented the GLP-1 response to glucose to a similar degree. Accordingly, metformin may modulate GLP-1 secretion via indirect mechanisms. As discussed earlier, the effects of metformin on bile acid resorption, glucose absorption and gut microbiota are likely to be relevant to GLP-1 secretion (Figure 1) [24].

That endogenous GLP-1 is central to the lowering of glucose by metformin is strongly supported by the abrogation of glucose-lowering when metformin is co-administrated with the GLP-1 receptor antagonist, exendin-9-39, in both rodents and humans [21,75]. While primarily known for its glucose-lowering capacity, GLP-1 has numerous extra-glycemic effects on multiple organ systems, including the cardiovascular system [60]. For example, in paucine models of myocardial infarction and heart failure, both GLP-1 and the long-acting GLP-1 receptor agonist, liraglutide, improved left ventricular contractile function and prolonged survival [76,77]. GLP-1 receptor activation exerts a cytoprotective effect in human umbilical vein endothelial cells and maintains endothelial cell function in T2D [78,79]. Furthermore, exogenous administration of GLP-1 attenuates the fall in postprandial blood pressure [80], the potential clinical significance of which will be discussed. Numerous cardioprotective effects of GLP-1 in humans have been demonstrated by GLP-1 receptor agonists, including weight loss, a mild reduction in systolic blood pressure, independent of weight loss, reduction in chronic inflammation, renal protection and attenuation of the fall in postprandial blood pressure [81,82]. Given that GLP-1 receptor agonists have now been shown to reduce rates of cardiovascular mortality, non-fatal myocardial infarction and non-fatal stroke, it is likely that these cardiovascular benefits are mediated by GLP-1 [83]. Whether GLP-1 is also implicated in the cardiovascular effects of metformin remains to be established.

### 2.5. Slowing of Gastric Emptying

Gastric emptying, the process by which the stomach empties its contents into the duodenum, exhibits a substantial inter-individual (in the range of 1–4 kcal/min in health), but much smaller intra-individual, variation [84]. There is now wide appreciation that gastric emptying represents a major determinant of postprandial glycemic excursions. In health, gastric emptying exhibits a direct relationship with the “early” glycemic response to oral glucose (i.e., the rise in blood glucose at 30 and 60 min), but an inverse relationship with “late” glycemia (i.e., blood glucose at 120 min). In individuals with impaired glucose tolerance or T2D, both early and late glycemic responses to carbohydrate tend to be positively related to the rate of gastric emptying. As a result, the overall glycemic response to oral glucose (incremental area under the curve over 120 min), correlates directly with the emptying rate in individuals with impaired glucose tolerance, but not in health, this difference is attributable to delayed insulin release and impaired insulin sensitivity with impaired glucose tolerance [85]. It is therefore logical to slow gastric emptying to reduce postprandial glycemic excursions in patients with T2D. Indeed, slowing gastric emptying underpins the postprandial glucose-lowering effect of both short- and, probably to a lesser extent, long-acting GLP-1 receptor agonists [86,87]. As discussed, postprandial hyperglycemia represents an independent risk factor for cardiovascular events. To this end, variations in the rate of gastric emptying may potentially influence cardiovascular health. A predilection for cardiovascular events has been associated with abnormally rapid or delayed gastric emptying in T2D [88]. Relatively more rapid gastric emptying has been associated with a marked fall in postprandial blood pressure in both older individuals with or without T2D [89]. Dietary and pharmacological interventions that slow gastric emptying are effective in attenuating the fall in blood pressure after a meal [64,90,91]. Metformin consistently slows gastric emptying in animal studies [92,93], and we recently showed in T2DM patients that metformin, delivered intraduodenally, slowed the gastric emptying of a liquid meal, without induction of nausea [74]. This effect may reflect augmented plasma levels of GLP-1, with potential contributions from glucagon-like peptide-2 and peptide-YY, co-released with GLP-1 from L-cells, the plasma levels of which are also enhanced by metformin administration [22,94,95,96]. The relative importance of gastric emptying to postprandial glucose-lowering by metformin remains to be established by longer-term studies. Of note, pathological delay in gastric emptying in T2D, i.e., gastroparesis, has been associated in patients with upper gastrointestinal symptoms, with major cardiovascular events [97], albeit without an association with mortality in other series [98]. Potentially, delaying gastric emptying carries therapeutic benefit generally in T2D but, when mediated pathologically by T2D, patients are at an increased risk of cardiovascular mortality by the impact of underlying autonomic neuropathy.

### 2.6. Attenuation of Postprandial Hypotension

Postprandial hypotension, defined as a fall in systolic blood pressure >20 mmHg within 2 h of a meal, is an important clinical phenomenon, but has been inappropriately underappreciated by clinicians. It occurs frequently (~20–44%) in apparently healthy elderly individuals and patients with T2D, and predisposes to syncope, falls, nausea and visual disturbance [99]. It also increases the risk of coronary events, stroke and mortality [100]. Hitherto, there has been a lack of established management options.

The fall in postprandial blood pressure reflects an inadequate cardiovascular compensation to meal-induced splanchnic blood pooling. The magnitude of the reduction is, therefore, related to the complex interactions between nutrients and the gastrointestinal tract. Several factors are now recognized to be important, including the rate of nutrient delivery to the small intestine (i.e., gastric emptying) and subsequent nutrient absorption, meal composition and neurohormonal responses [99]. There exists a nonlinear relationship between small intestinal nutrient delivery and the magnitude in systolic blood pressure decline, such that there appears to be a threshold—likely between 1 and 3 kcal/min—above which nutrient delivery can induce a hypotensive response [101]. This threshold probably relates to the degree of intestinal nutrient stimulation required to induce a splanchnic vasodilator response, resulting in splanchnic blood pooling and reduced systemic blood pressure, and may relate to the release of vasoactive peptides. If the ingested meal contains more complex macronutrients (such as starch rather than glucose), this delays the absorption of nutrients and their interaction with the gut, associated with attenuation of the fall in postprandial blood pressure [102].

Multiple neurohormonal mechanisms have been implicated in the postprandial hypotensive response. Meal ingestion activates the sympathetic nervous system via the “gastrovascular reflex,” involving stimulation of noradrenaline by gastric distension, leading to increases in heart rate, left ventricular contractility and systemic vasoconstriction. In older individuals, and those with postprandial hypotension, this response is often blunted [99]. A number of vasoactive gastrointestinal hormones are implicated in the pathogenesis of postprandial hypotension, including bradykinin and substance P, while others, such as GLP-1, are thought to be protective [99]. Infusion of GLP-1 increases blood pressure and heart rate in rodents [103]. While most human trials involving administration of GLP-1 receptor agonists to patients with T2D reported reductions in blood pressure, these studies failed to discriminate between effects on fasting and postprandial blood pressure [60]. A “physiological” dose of GLP-1 (0.9 pmolkg^−1^min^−1^) has been shown to attenuate the hypotensive response to an enteral glucose infusion in healthy older human subjects [80]. Moreover, when the GLP-1 receptor agonist, exenatide, was administered with a concurrent intraduodenal glucose infusion in T2D, blood pressure and heart rate increased rather than decreased, when compared to control [82].

**Figure 2:**
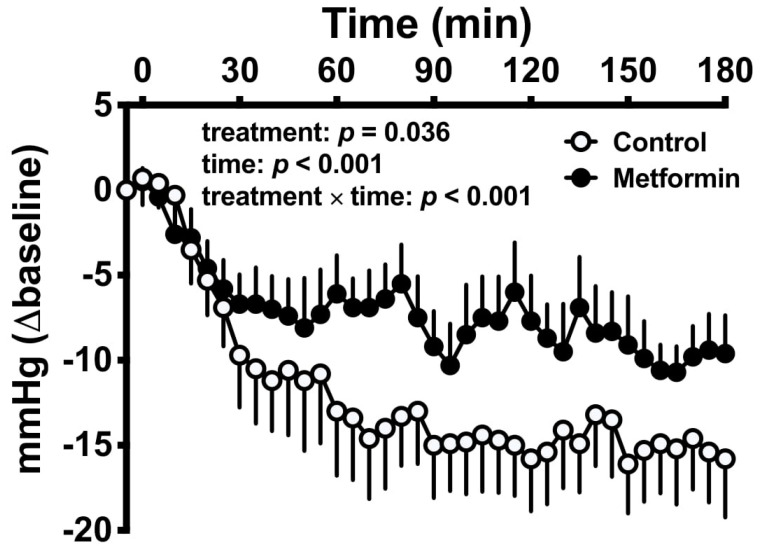
Metformin attenuates the fall in blood pressure induced by oral glucose [23]. Attenuation of the fall in systolic blood pressure after intraduodenal administration of metformin or placebo and a 50 g oral glucose drink in T2D. Two-factor repeated measures ANOVA, with treatment and time as factors, was used to determine statistical significance. Results of ANOVA are reported as P values for differences by treatment, differences over time and differences because of the interaction of the two factors. Data are mean values ± standard error of the mean.

Metformin impacts multiple determinants of the postprandial blood pressure response, by slowing gastric emptying and inhibiting glucose absorption, enhancing GLP-1 secretion, and potentially—based on rodent models—increasing noradrenaline secretion to enhance left ventricular function (Figure 1) [23,59,104,105]. It is perhaps surprising that metformin was only recently shown to attenuate the fall in blood pressure induced by an oral glucose drink in patients with T2D. In this study, a clinically therapeutic dose (1 g) of metformin was infused directly into the duodenum, thereby circumventing the potential influence of gastric emptying on small intestinal exposure to metformin (Figure 2) [23]. Further studies are required to determine whether this effect is sustained with ongoing metformin use.

## 3. Conclusions

Metformin exerts heterogenous gastrointestinal effects, including suppression of intestinal bile acid resorption, modulation of the gut microbiota, reduction in the rate of glucose absorption, enhancement of GLP-1 secretion and action, and slowing of gastric emptying. While it is increasingly appreciated that these actions are fundamental to its glucose-lowering effect, their potential influence on the cardiovascular outcomes of metformin therapy has received little attention. In an acute setting, metformin has been shown to attenuate the postprandial fall in blood pressure substantially in T2D, in association with the slowing of gastric emptying and stimulation of GLP-1 secretion. However, the definition of mechanisms underlying the cardiovascular benefits of metformin are limited by inconsistency in the outcomes of animal and human studies, and the current lack of clear associations between these mechanisms and cardiovascular health in the longitudinal studies of T2D patients. Mechanistic insights into the relevance of the aforementioned gastrointestinal mechanisms to the cardiovascular outcomes of metformin in longer-term studies may well refine the clinical application of an “old” antidiabetic drug.
